# Association of candidate pharmacogenetic markers with platinum-induced ototoxicity: PanCareLIFE dataset

**DOI:** 10.1016/j.dib.2020.106227

**Published:** 2020-08-24

**Authors:** Thorsten Langer, Eva Clemens, Linda Broer, Lara Maier, André G. Uitterlinden, Andrica C.H. de Vries, Martine van Grotel, Saskia F.M. Pluijm, Harald Binder, Benjamin Mayer, Annika von dem Knesebeck, Julianne Byrne, Eline van Dulmen-den Broeder, Marco Crocco, Desiree Grabow, Peter Kaatsch, Melanie Kaiser, Claudia Spix, Line Kenborg, Jeanette F. Winther, Catherine Rechnitzer, Henrik Hasle, Tomas Kepak, Anne-Lotte F. van der Kooi, Leontien C. Kremer, Jarmila Kruseova, Stefan Bielack, Benjamin Sorg, Stefanie Hecker-Nolting, Claudia E. Kuehni, Marc Ansari, Martin Kompis, Heleen J. van der Pal, Ross Parfitt, Dirk Deuster, Peter Matulat, Amelie Tillmanns, Wim J.E. Tissing, Jörn D. Beck, Susanne Elsner, Antoinette am Zehnhoff-Dinnesen, Marry M. van den Heuvel-Eibrink, Oliver Zolk, PanCareLIFE consortium

**Affiliations:** a Department of Pediatric Oncology and Hematology, University Hospital for Children and Adolescents, Lübeck, Germany; b Princess Máxima Center for Pediatric Oncology, Utrecht, The Netherlands; c Department of Pediatric Oncology, Erasmus MC – Sophia Children's Hospital, Rotterdam, The Netherlands; d Department of Internal Medicine, Erasmus Medical Center, Rotterdam, The Netherlands; e Institute of Pharmacology of Natural Products and Clinical Pharmacology, Ulm University Medical Center, Ulm, Germany; f German Childhood Cancer Registry, Institute of Medical Biostatistics, Epidemiology and Informatics, University Medical Center of the Johannes Gutenberg University Mainz, Mainz, Germany; g Institute of Medical Biometry and Statistics, Faculty of Medicine and Medical Center, University of Freiburg, Freiburg, Germany; h Institute of Epidemiology and Medical Biometry, University of Ulm, Ulm, Germany; i Boyne Research Institute, Drogheda, Ireland; j Department of Pediatric Hematology and Oncology, VU Medical Center, Amsterdam, The Netherlands; k Department of Neurooncology, Istituto Giannina Gaslini, Genova, Italy; l Danish Cancer Society Research Center, Childhood Cancer Research Group, Copenhagen, Denmark; m Department of Clinical Medicine, Faculty of Health, Aarhus University, Aarhus, Denmark; n Copenhagen University Hospital Rigshospitalet, Department of Pediatrics and Adolescent Medicine, Copenhagen, Denmark; o Aarhus University Hospital, Department of Pediatrics, Aarhus University Hospital, Aarhus, Denmark; p University Hospital Brno, Brno, Czech Republic, & International Clinical Research Center (FNUSA-ICRC), Brno, Czech Republic; q Department of Obstetrics and Gynecology, Erasmus MC – Sophia Children's Hospital, The Netherlands; r Department of Pediatric Oncology, Academic Medical Center Amsterdam, Amsterdam, The Netherlands; s Department of Children Hemato-Oncology, Motol University Hospital Prague, Prague, Czech Republic; t Department of Pediatric Oncology, Hematology, Immunology, Stuttgart Cancer Center, Olgahospital, Stuttgart, Germany; u Institute of Social and Preventive Medicine, University of Bern, Bern, Switzerland; v Paediatric Oncology, Dept. of Paediatrics, Inselspital, University of Bern, Switzerland; w Department of Pediatrics, Oncology and Hematology Unit, University Hospital of Geneva, Cansearch Research Laboratory, Geneva University, Switzerland; x Department of Otolaryngology, Head and Neck Surgery, Inselspital, University of Berne; y Department of Phoniatrics and Pedaudiology, University Hospital Münster, Westphalian Wilhelm University, Münster, Germany; z Department of Pediatric Oncology, University of Groningen, University Medical Center Groningen, Groningen, The Netherlands; # Hospital for Children and Adolescents, University of Erlangen-Nuremberg, Erlangen, Germany; $ Institute for Social Medicine and Epidemiology, University of Lübeck, Lübeck, Germany; % Institute of Clinical Pharmacology, Immanuel Klinik Rüdersdorf, Brandenburg Medical School Theodor Fontane, Germany

**Keywords:** Cancer survivors, Childhood cancer, Cisplatin: carboplatin, Drug-induced ototoxicity, Adverse drug reaction, Pharmacogenetics, Genetic markers, Multicenter cohort study

## Abstract

Genetic association studies suggest a genetic predisposition for cisplatin-induced ototoxicity. Among other candidate genes, thiopurine methyltransferase (*TPMT*) is considered a critical gene for susceptibility to cisplatin-induced hearing loss in a pharmacogenetic guideline. The PanCareLIFE cross-sectional cohort study evaluated the genetic associations in a large pan-European population and assessed the diagnostic accuracy of the genetic markers. 1,112 pediatric cancer survivors who had provided biomaterial for genotyping were screened for participation in the pharmacogenetic association study. 900 participants qualified for inclusion. Based on the assessment of original audiograms, patients were assigned to three phenotype categories: no, minor, and clinically relevant hearing loss. Fourteen variants in eleven candidate genes (*ABCC3, OTOS, TPMT, SLC22A2, NFE2L2, SLC16A5, LRP2, GSTP1, SOD2, WFS1,* and *ACYP2*) were genotyped. The genotype and phenotype data represent a resource for conducting meta-analyses to derive a more precise pooled estimate of the effects of genes on the risk of hearing loss due to platinum treatment.

**Specifications Table**

**Table:** 

Subject	Oncology
Specific subject area	Late effects of cancer treatment; pharmacogenetics
Type of data	Table
How data were acquired	Clinical data: from servers for the electronic medical records and registries and by manual review of patient medical charts. Genotype data: Applied Biosystems 7500 FastReal-Time PCR System
Data format	Raw Analyzed Filtered
Parameters for data collection	Genotype data: Applied Biosystems 7500 Real-Time PCR System Sequence Detection Software v1.4, automatic genotype call algorithm.
Description of data collection	Clinical data: Data for enrolled patients from medical records were entered into a trial-specific database hosted at the German Childhood Cancer Registry. Genotype data: Genomic DNA was isolated from EDTA blood samples or saliva samples and tested for quality. Samples were genotyped for 14 SNPs by TaqMan SNP genotyping using predesigned primers and probes (Applied Biosystems, Foster City, CA, USA). Audiological Classification and Phenotype data: Patients were assigned to one of three phenotypes based on the grading of the audiograms according to the Münster Classification.
Data source location	PanCareLIFE Data Center, German Childhood Cancer Registry Mainz Germany
Data accessibility	With the article
Related research article	Thorsten Langer, Eva Clemens, Linda Broer, Lara Maier, André G. Uitterlinden, Andrica C. H. de Vries, Martine van Grotel, Saskia F.M. Pluijm, Harald Binder, Benjamin Mayer, Annika von dem Knesebeck, Julianne Byrne, Eline van Dulmen-den Broeder, Marco Crocco, Desiree Grabow, Peter Kaatsch, Melanie Kaiser, Claudia Spix, Line Kenborg, Jeanette F. Winther, Catherine Rechnitzer, Henrik Hasle, Tomas Kepak, Anne-Lotte F. van der Kooi, Leontien C. Kremer, Jarmila Kruseova, Stefan Bielack, Benjamin Sorg, Stefanie Hecker-Nolting, Claudia E. Kuehni, Marc Ansari, Martin Kompis, Heleen van der Pal, Ross Parfitt, Dirk Deuster, Peter Matulat, Amelie Tillmanns, Wim J. E. Tissing, Jörn D. Beck, Susanne Elsner, Antoinette am Zehnhoff-Dinnesen, Marry M. van den Heuvel-Eibrink, and Oliver Zolk, on behalf of the PanCareLIFE consortium Usefulness of current candidate genetic markers to identify childhood cancer patients at risk for platinum-induced ototoxicity: results of the European PanCareLIFE cohort study. Eur. J. Cancer. In Press.

**Value of the Data**

• This database describes genotypes of 14 candidate SNPs for cisplatin-induced ototoxicity in a large Pan-European cohort of pediatric cancer survivors treated with platinum.
• Epidemiologists interested in the frequency of platinum-induced ototoxicity as well as developers of long-term follow-up guidelines for survivors of childhood, adolescent, and young adult cancers may benefit from these data.
• The genotype and phenotype data represent a resource for conducting meta-analyses to derive a more precise pooled estimate of the effects of genes on the risk of hearing loss due to platinum treatment.

## 1 Data Description

Table 1 summarize the genotype and allele frequencies of the study population, stratified according to the hearing loss phenotype.

**Table 1:** Number and frequency of genotypes and alleles for each single nucleotide polymorphism (SNP) according to audiological phenotypes. The last column shows the P-values of the Hardy-Weinberg equilibrium (HWE) χ2 tests of the total cohort (n = 900).

SNP	Genotype/ allele	Number of genotypes / alleles			Frequency	HWE Chi-squared test P value
		no hearing loss (n = 222)	minor hearing loss (n = 481)	clinically relevant hearing loss (n = 197)	Total cohort (n = 900)	
*ABCC3* rs1051640	A/A	152	337	131	0.665	0.85
	A/G	64	128	61	0.310	
	G/G	6	16	5	0.025	
	A	368	802	323	0.820	
	G	76	160	71	0.180	
*ACYP2* rs1872328	G/G	204	453	185	0.939	0.32
	G/A	18	28	12	0.061	
	A/A	0	0	0	0.000	
	G	426	934	382	0.970	
	A	18	28	12	0.030	
*GSTP1* rs1695	A/A	95	221	89	0.452	0.58
	A/G	104	212	76	0.386	
	G/G	23	48	32	0.162	
	A	294	654	254	0.645	
	G	150	308	140	0.355	
*LRP2* rs2075252	T/T	15	32	12	0.061	0.26
	T/C	79	165	74	0.376	
	C/C	128	284	111	0.563	
	T	109	229	98	0.249	
	C	335	733	296	0.751	
*NFE2L2* rs6721961	T/T	2	7	3	0.015	0.82
	T/G	51	89	50	0.254	
	G/G	169	385	144	0.731	
	T	55	103	56	0.142	
	G	389	859	338	0.858	
*OTOS* rs2291767	T/T	215	453	188	0.954	0.55
	T/C	7	27	9	0.046	
	C/C	0	1	0	0.000	
	T	437	933	385	0.977	
	C	7	29	9	0.023	
*SLC16A5* rs4788863	T/T	16	37	12	0.061	0.27
	T/C	97	199	82	0.416	
	C/C	109	245	103	0.523	
	T	129	273	106	0.269	
	C	315	689	288	0.731	
*SLC22A2* rs316019	A/A	3	3	2	0.010	0.43
	A/C	44	85	47	0.239	
	C/C	175	393	148	0.751	
	A	50	91	51	0.129	
	C	394	871	343	0.871	
*SOD2* rs4880	A/A	56	106	40	0.203	0.36
	A/G	109	249	105	0.533	
	G/G	57	126	52	0.264	
	A	221	461	185	0.470	
	G	223	501	209	0.530	
*TPMT* rs12201199	A/A	205	434	174	0.883	0.6
	A/T	17	44	23	0.117	
	T/T	0	3	0	0.000	
	A	427	912	371	0.942	
	T	17	50	23	0.058	
*TPMT* rs1142345	T/T	211	452	182	0.924	0.34
	T/C	11	29	15	0.076	
	C/C	0	0	0	0.000	
	T	433	933	379	0.962	
	C	11	29	15	0.038	
*TPMT* rs1800460	C/C	212	454	184	0.934	0.39
	C/T	10	27	13	0.066	
	T/T	0	0	0	0.000	
	C	434	935	381	0.967	
	T	10	27	13	0.033	
*TPMT* rs1800462	C/C	220	481	196	0.995	0.96
	C/G	2	0	1	0.005	
	G/G	0	0	0	0.000	
	C	442	962	393	0.997	
	G	2	0	1	0.003	
*WFS1* rs62283056	G/G	138	307	124	0.629	0.97
	G/C	79	149	65	0.330	
	C/C	5	25	8	0.041	
	G	355	763	313	0.794	
	C	89	199	81	0.206	

The supplementary Table 1 shows demographic and clinical variables at a patient-level of the total cohort (n = 900). Variables are described as follows:

ID – Unique identification number assigned to each patient who was included in the analyses.
RX – Cranial radiation (0) or no cranial radiation (1)
SEX – Male (1) or female (2)
PHENO – audiological phenotype: no hearing loss (0), minor hearing loss (1), clinically relevant hearing loss (2). Patients were assigned to the respective audiological phenotype based on the post-treatment audiograms of the patients, which were graded according to the Münster Classification. A detailed description of the phenotyping method is given below.
AGE – age at start of platinum treatment: < = 5 years (1),]5 years; 10 years] (2),]10 years; 15 years] (3), >15 years (4).
CISPLATIN – Cumulative dose of cisplatin (mg)
CARBOPLATIN – Cumulative dose of carboplatin (mg)
DIAGNOSIS – the cancer diagnosis

The supplementary Table 2 shows the genotype data at a patient-level of the total cohort (n = 900). Variables are described as follows:

SAMPLE_ID – Unique identification number assigned to the gDNA sample of each patient included in the analyses.
ABCC3_rs1051640 – the rs1051640 genotype: A/A (0), A/G (1), G/G (2)
OTOS_rs2291767 – the rs2291767 genotype: T/T (0), T/C (1), C/C (2)
TPMT_rs12201199 – the rs12201199 genotype: A/A (0), A/T (1), T/T (2)
TPMT_rs1142345 – the rs1142345 genotype: T/T (0), T/C (1), C/C (2)
TPMT_rs1800460 – the rs1800460 genotype: C/C (0), C/T (1), T/T (2)
TPMT_rs1800462 – the rs1800462 genotype: C/C (0), C/G (1), G/G (2)
SLC22A2_rs316019 – the rs316019 genotype: A/A (0), A/C (1), C/C (2)
NFE2L2_rs6721961 – the rs6721961 genotype: T/T (0), T/G (1), G/G (2)
WFS1_rs62283056 – the rs62283056 genotype: G/G (0), G/C (1), C/C (2)
SLC16A5_rs4788863 –the rs4788863 genotype: T/T (0), T/C (1), C/C (2)
LRP2_rs2075252 – the rs2075252 genotype: T/T (0), T/C (1), C/C (2)
GSTP1_rs1695 – the rs1695 genotype: A/A (0), A/G (1), G/G (2)
SOD2_rs4880 – the rs4880 genotype: A/A (0), A/G (1), G/G (2)
ACYP2_rs1872328 – the rs1872328 genotype: G/G (0), G/A (1), A/A (2)

## 2 Experimental Design, Materials and Methods

### 2.1 Study design and participants

Background and methods of the European multicenter PanCareLIFE study have been described previously [1], [2], [3]. Patients were enrolled after approval was obtained from local review boards and written informed consent was obtained from patients, parents or legal guardians. Participants were enrolled both retrospectively and prospectively (i.e., chemotherapy was started and finished during the 5-year term of PanCareLIFE). Eligibility criteria were: 1) age at diagnosis <19 years, 2) treatment with cisplatin, carboplatin or both, 3) at least one pure tone audiometry within 5 years after the end of chemotherapy. Exclusion criteria were: 1) non-consent and 2) hearing loss before the start of platinum treatment. Patients of this larger ototoxicity cohort participated in the pharmacogenetic study if there was additional consent for the genetic analyses and biomaterial was provided.

### 2.2 Genotyping

Biosamples were sent to the PanCareLIFE genotyping center. Genomic DNA (gDNA) was isolated from EDTA blood samples with a QIAamp DNA Blood Kit (Qiagen, Hilden, Germany) or from saliva samples (Oragene DNA collection kit, DNA Genotec, Ottawa, ON, Canada) using the prepIT L2P reagent (DNA Genotec, Ottawa, ON, Canada). All gDNA samples isolated were tested for quality (A260/A280 ratio of >1.9 and agarose gele electrophoresis) before any further work on DNA analysis. Samples were genotyped for 14 SNPs by TaqMan SNP genotyping using predesigned primers and probes (Applied Biosystems, Foster City, CA, USA). In order not to lose too much statistical power, the number of candidate genes was limited to 11 with one SNP each except for TPMT, for which 4 SNPs were examined. The candidate SNPs were selected on the basis of the available evidence of association, taking into account the sample size of the discovery cohort and the effect size. The following SNPs were investigated: rs1872328 (*ACYP2*), rs2075252 (*LRP2*), rs6721961 (*NFE2L2*), rs2291767 (*OTOS*), rs62283056 (*WFS1*), rs12201199 (*TPMT*), rs1142345 (*TPMT*), rs1800460 (*TPMT*), rs1800462 (*TPMT*), rs4880 (*SOD2*), rs316019 (*SLC22A2*), rs1695 (*GSTP1*), rs1051640 (*ABCC3*), and rs4788863 (*SLC16A5*).

Laboratory assistants were blinded to the audiological phenotype of the patients. Multiple positive and negative controls and replicate samples were included in the genotyping assays and plates. No genotype discordance of replicate samples was observed. Ten samples were finally excluded due to genotype call rate per sample <100%.

### 2.3 Audiological classification and phenotyping

All audiograms were independently rated by two reviewers for hearing loss according to the Münster classification [4,5]. Audiograms had to meet the following minimum requirements: frequencies include at least 2 or 3 kHz, 4 kHz, and 6 or 8 kHz (air-conduction), demonstrate no conductive hearing loss, absence of significant test artifacts (e.g., atypical air-bone configuration).

Thereafter, two pediatric audiologists independently assessed the kinetic course of hearing loss for each patient. The minimum data requirement for phenotype assessment included the availability of a normal pre-treatment audiogram or a normal audiogram before the third platinum cycle and at least one post-treatment audiogram within 15 months after the last chemotherapy cycle. Sound field audiometry was also accepted if ear-specific pure-tone audiometry was subsequently performed. Three phenotype groups were defined as follows: no hearing loss, minor hearing loss, and clinically-relevant hearing loss at the end of treatment. Patients were assigned to the no hearing loss group if post-treatment audiograms were exclusively Münster class 0. Patients were also assigned to the group without hearing loss if post-treatment audiograms were almost exclusively graded as Münster class 0, no audiogram was classified as Münster >1, and the Münster class 1 audiogram was followed by a Münster class 0 audiogram. Patients were assigned to the clinically-relevant hearing loss group if follow-up audiograms indicated hearing loss of at least Münster class 2b. All other patients were classified as part of the minor hearing loss group. Inter-rater agreement was >95%. After completion, all cases that had been phenotyped differently by the two pediatric audiologists were discussed between them and an agreement was made.

## Ethics Statement

The PanCareLIFE study has been approved by the local ethics committees: Kantonale Ethikkommission Bern, 362/2015; Comitate Etico Regionale, 507REG2014; Ethical Committee University Hospital Brno, June 11, 2016; Ethics Committee Fakultni Nemocnice v Motole, Prague; De Videnskabsetiske Komiteer Region Hovedstaden, H-1-2014-125; Ethikkommission Medizinische Universität Graz, 27-015 ex 14/15; Ethikkommission der Universität Ulm, 160/17; Ethikkommission der Universität zu Lübeck, 14/181; Ethik-Kommission der Ärztekammer Westfalen-Lippe und der Westfälischen Wilhelms-Universität Münster, 2014-619; Medische Ethische Toetsings Commissie Erasmus MC; Medisch Ethische Toetsingscommissie, 2015_202. The informed consent of the patient (if adult) or his/her legal representative has been obtained.

## Declaration of Competing Interest

The authors declare that they have no known competing financial interests or personal relationships which have, or could be perceived to have, influenced the work reported in this article.

## Appendix Supplementary materials

Image, application 1

Image, application 2
